# Ethyl 2-(1,3-benzodioxol-5-yl)-1-[3-(1*H*-imidazol-1-yl)prop­yl]-1*H*-benzimidazole-5-carboxyl­ate

**DOI:** 10.1107/S1600536811054572

**Published:** 2011-12-23

**Authors:** Yeong Keng Yoon, Mohamed Ashraf Ali, Tan Soo Choon, Safra Izuani Jama Asik, Ibrahim Abdul Razak

**Affiliations:** a Institute for Research in Molecular Medicine, Universiti Sains Malaysia, Minden 11800, Penang, Malaysia; b School of Physics, Universiti Sains Malaysia, 11800 USM, Penang, Malaysia

## Abstract

In the title compound, C_23_H_22_N_4_O_4_, the essentially planar [maximum deviation = 0.022 (1) Å] benzimidazole ring system forms dihedral angles of 86.16 (7) and 37.38 (6)°, respectively, with the imidazole and benzene rings. The dioxolane ring adopts an envelope conformation with the methyl­ene C atom at the flap. In the crystal, C—H⋯O and C—H⋯N inter­actions link the mol­ecules into a ribbon along the *a* axis. The crystal packing is further stabilized by weak π–π stacking inter­actions [centroid–centroid distances = 3.5954 (8) and 3.7134 (8) Å] and C—H⋯π inter­actions.

## Related literature

For the biological activity of benzimidazole derivatives, see: Grassmann *et al.* (2002 ▶); Demirayak *et al.* (2002 ▶). For puckering parameters, see: Cremer & Pople (1975 ▶). For stability of the temperature controller used in the data collection, see: Cosier & Glazer (1986 ▶). For a related structure, see: Yoon *et al.* (2011 ▶).
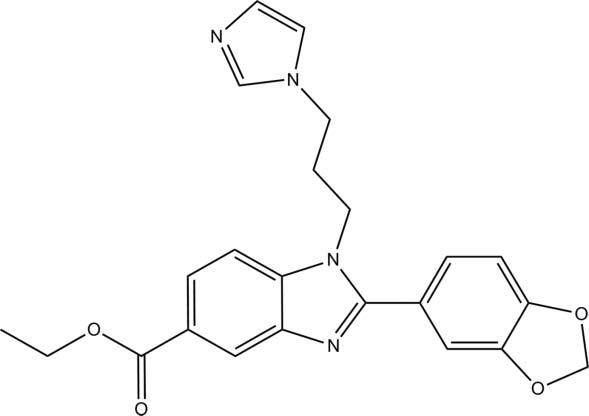


## Experimental

### 

#### Crystal data

C_23_H_22_N_4_O_4_
*M* *_r_* = 418.45
Orthorhombic, 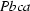

*a* = 15.8554 (2) Å
*b* = 15.3988 (2) Å
*c* = 16.2292 (2) Å
*V* = 3962.43 (9) Å^3^
*Z* = 8
Mo *K*α radiation
μ = 0.10 mm^−1^
*T* = 100 K
0.42 × 0.28 × 0.20 mm

#### Data collection

Bruker SMART APEXII CCD area-detector diffractometer
Absorption correction: multi-scan (*SADABS*; Bruker, 2009 ▶) *T*_min_ = 0.960, *T*_max_ = 0.981
39067 measured reflections
5782 independent reflections
4429 reflections with *I* > 2σ(*I*)
*R*_int_ = 0.054

#### Refinement

*R*[*F* ^2^ > 2σ(*F* ^2^)] = 0.051
*wR*(*F* ^2^) = 0.126
*S* = 1.06
5782 reflections
280 parameters
H-atom parameters constrained
Δρ_max_ = 0.42 e Å^−3^
Δρ_min_ = −0.26 e Å^−3^

### 

Data collection: *APEX2* (Bruker, 2009 ▶); cell refinement: *SAINT* (Bruker, 2009 ▶); data reduction: *SAINT*; program(s) used to solve structure: *SHELXTL* (Sheldrick, 2008 ▶); program(s) used to refine structure: *SHELXTL*; molecular graphics: *SHELXTL*; software used to prepare material for publication: *SHELXTL* and *PLATON* (Spek, 2009 ▶).

## Supplementary Material

Crystal structure: contains datablock(s) global, I. DOI: 10.1107/S1600536811054572/is5032sup1.cif

Structure factors: contains datablock(s) I. DOI: 10.1107/S1600536811054572/is5032Isup2.hkl

Supplementary material file. DOI: 10.1107/S1600536811054572/is5032Isup3.cml

Additional supplementary materials:  crystallographic information; 3D view; checkCIF report

## Figures and Tables

**Table 1:** Hydrogen-bond geometry (Å, °) *Cg*1 and *Cg*4 are the centroids of C11/C12/O3/C23/O4 and C1–C6 rings, respectively.

*D*—H⋯*A*	*D*—H	H⋯*A*	*D*⋯*A*	*D*—H⋯*A*
C2—H2*A*⋯O3^i^	0.95	2.48	3.3922 (17)	162
C15—H15*B*⋯O4^ii^	0.99	2.49	3.213 (2)	130
C23—H23*A*⋯N4^iii^	0.99	2.43	3.379 (2)	159
C10—H10*A*⋯*Cg*4^iv^	0.95	2.65	3.3005 (16)	126
C16—H16*C*⋯*Cg*1^v^	0.98	2.91	3.7282 (19)	142

Symmetry codes: (i) 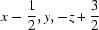; (ii) 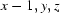; (iii) 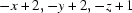; (iv) 
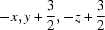
; (v) 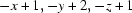.

**Fig. 1.**
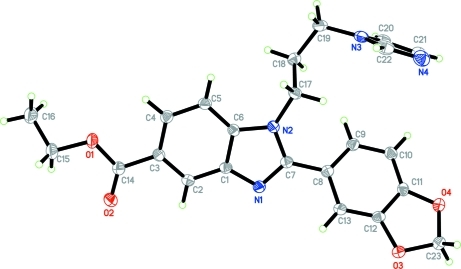
The structure of the title compound, showing 50% probability displacement ellipsoids. Hydrogen atoms are shown as spheres of arbitrary radius.

**Fig. 2.**
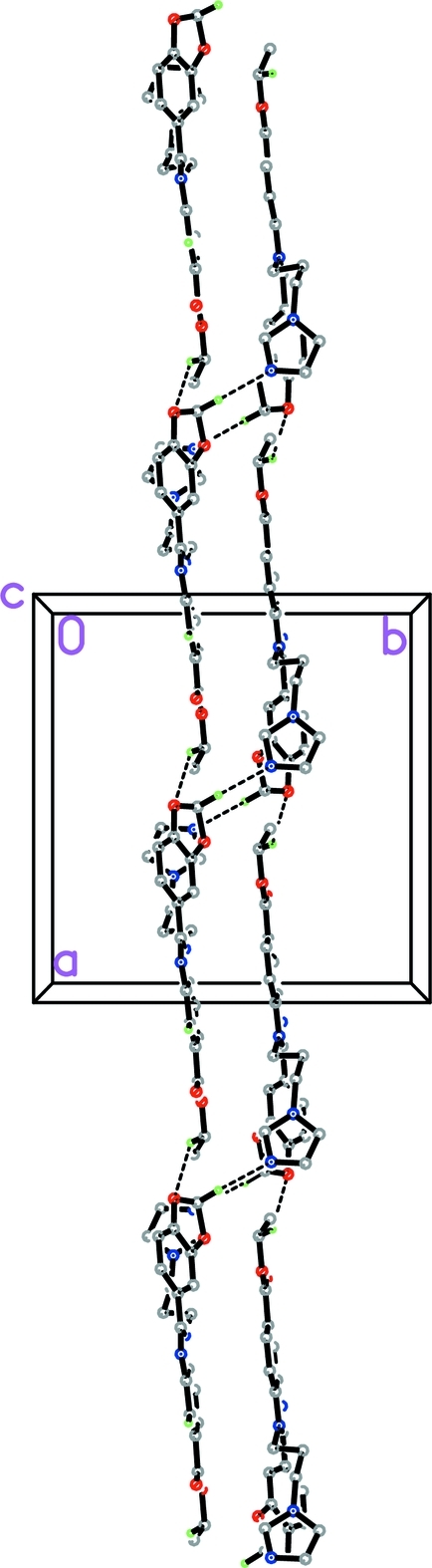
The crystal packing, viewed along the c-axis, showing the molecules are connected into ribbon along a axis. Hydrogen bonds are shown as dashed lines.

## supplementary crystallographic information

### Comment

Benzimidazole derivatives are of wide interest because of their diverse biological activities and various clinical applications. In particular, 2-substituted benzimidazoles have been proven as effective drug leads, thus generating pharmacological interests (Grassmann *et al.*, 2002; Demirayak *et al.*, 2002). As part of our ongoing structural studies of benzimidazole derivatives (Yoon *et al.*, 2011), we now report the structure of the title compound.

Fig. 1 shows the molecular structure of the title compound. The benzimidazole (N1–N2/C1–C7) ring is approximately planar with a maximum deviation of 0.022 (1) Å for atoms C6 and C7. The mean plane through this ring forms dihedral angles of 86.16 (7) and 37.38 (6)° with the mean plane through the imidazole (N3/N4/C20–C22) and benzene (C8–C13) rings, respectively. The dioxolane (O3/O4/C11/C12/C23) ring adopts an envelope conformation with puckering parameters Q = 0.1209 (14) Å and φ = 138.2 (7)° with atom C23 at the flap (Cremer & Pople, 1975).

In the crystal packing of (Fig. 2), intermolecular C2—H2A···O3(*x* - 1/2, *y*, -*z* + 3/2), C15—H15B···O4(-1 + *x*, *y*, *z*) and C23—H23A···N4(2 - *x*, 2 - *y*, 1 - *z*) interactions form the molecules into ribbon stacked along the *a*-axis. π–π interactions are observed within the benzimidazole ring system between the imidazole (N1/N2/C1/C6–C7; centroid *Cg*2) and benzene, (C1–C6; centroid *Cg*4) rings with a *Cg*2···*Cg*4(1 - *x* , 2 - *y*, 1 - *z*) distance of 3.5954 (8) Å and between the benzene, (C1–C6; centroid *Cg*4) rings with a *Cg*4···*Cg*4 (1 - *x*, 2 - *y*, 1 - *z*) distance of 3.7134 (8) Å. The crystal packing are further stabilized by weak C—H···π interactions (Table 1) involving the benzene ring of the benzimidazole moiety and the dioxolane ring with the distances of 3.3005 (16) and 3.7282 (19) Å, respectively.

### Experimental

Ethyl-4-[3-(1*H*-imidazol-1-yl)propylamino]-3-aminobenzoate (0.84 mmol) and sodium metabisulfite adduct of piperonal (1.68 mmol) were dissolved in DMF. The reaction mixture was reflux at 130 °C for 2 hrs. After completion, the reaction mixture was diluted in ethyl acetate (20 mL) and washed with water (20 mL). The organic layer was collected, dried over Na_2_SO_4_ and the evaporated in vacuo to yield the product. The product was recrystallised from ethyl acetate.

### Refinement

All the H atoms positioned geometrically and refined using a riding model with C—H = 0.95–0.99 Å. The *U*_iso_ values were constrained to be 1.5*U*_eq_ (methyl-H atom) and 1.2*U*_eq_ (other H atoms). The rotating model group was applied for the methyl group.

### Crystal data

**Table:**

C_23_H_22_N_4_O_4_	*F*(000) = 1760
*M**_r_* = 418.45	*D*_x_ = 1.403 Mg m^−^^3^
Orthorhombic, *P**b**c**a*	Mo *K*α radiation, λ = 0.71073 Å
Hall symbol: -P 2ac 2ab	Cell parameters from 7948 reflections
*a* = 15.8554 (2) Å	θ = 2.2–29.9°
*b* = 15.3988 (2) Å	µ = 0.10 mm^−^^1^
*c* = 16.2292 (2) Å	*T* = 100 K
*V* = 3962.43 (9) Å^3^	Block, yellow
*Z* = 8	0.42 × 0.28 × 0.20 mm

### Data collection

**Table:**

Bruker SMART APEXII CCD area-detector diffractometer	5782 independent reflections
Radiation source: fine-focus sealed tube	4429 reflections with *I* > 2σ(*I*)
graphite	*R*_int_ = 0.054
φ and ω scans	θ_max_ = 30.0°, θ_min_ = 2.2°
Absorption correction: multi-scan (*SADABS*; Bruker, 2009)	*h* = −22→22
*T*_min_ = 0.960, *T*_max_ = 0.981	*k* = −17→21
39067 measured reflections	*l* = −22→22

### Refinement

**Table:**

Refinement on *F*^2^	Primary atom site location: structure-invariant direct methods
Least-squares matrix: full	Secondary atom site location: difference Fourier map
*R*[*F*^2^ > 2σ(*F*^2^)] = 0.051	Hydrogen site location: inferred from neighbouring sites
*wR*(*F*^2^) = 0.126	H-atom parameters constrained
*S* = 1.06	*w* = 1/[σ^2^(*F*_o_^2^) + (0.0539*P*)^2^ + 1.7654*P*] where *P* = (*F*_o_^2^ + 2*F*_c_^2^)/3
5782 reflections	(Δ/σ)_max_ = 0.001
280 parameters	Δρ_max_ = 0.42 e Å^−^^3^
0 restraints	Δρ_min_ = −0.26 e Å^−^^3^

### Special details

**Table:**

Experimental. The crystal was placed in the cold stream of an Oxford Cryosystems Cobra open-flow nitrogen cryostat (Cosier & Glazer, 1986) operating at 100.0 (1) K.
Geometry. All esds (except the esd in the dihedral angle between two l.s. planes) are estimated using the full covariance matrix. The cell esds are taken into account individually in the estimation of esds in distances, angles and torsion angles; correlations between esds in cell parameters are only used when they are defined by crystal symmetry. An approximate (isotropic) treatment of cell esds is used for estimating esds involving l.s. planes.
Refinement. Refinement of F^2^ against ALL reflections. The weighted R-factor wR and goodness of fit S are based on F^2^, conventional R-factors R are based on F, with F set to zero for negative F^2^. The threshold expression of F^2^ > 2sigma(F^2^) is used only for calculating R-factors(gt) etc. and is not relevant to the choice of reflections for refinement. R-factors based on F^2^ are statistically about twice as large as those based on F, and R- factors based on ALL data will be even larger.

### Fractional atomic coordinates and isotropic or equivalent isotropic displacement parameters (Å^2^)

**Table:**

	*x*	*y*	*z*	*U*_iso_*/*U*_eq_	
O1	0.21824 (6)	0.91984 (8)	0.49482 (7)	0.0282 (3)	
O2	0.25425 (7)	0.90679 (7)	0.62843 (7)	0.0271 (2)	
O3	0.89060 (6)	0.92843 (7)	0.71394 (6)	0.0239 (2)	
O4	0.98149 (6)	0.84912 (7)	0.63028 (7)	0.0263 (2)	
N1	0.58208 (7)	0.86631 (8)	0.59836 (7)	0.0194 (2)	
N2	0.61041 (7)	0.87485 (8)	0.46195 (7)	0.0187 (2)	
N3	0.79424 (8)	0.84083 (8)	0.26915 (7)	0.0225 (3)	
N4	0.91848 (9)	0.89721 (9)	0.30370 (9)	0.0296 (3)	
C1	0.50764 (8)	0.87928 (9)	0.55479 (8)	0.0182 (3)	
C2	0.42516 (8)	0.88928 (9)	0.58316 (9)	0.0190 (3)	
H2A	0.4125	0.8865	0.6403	0.023*	
C3	0.36188 (8)	0.90350 (9)	0.52518 (9)	0.0191 (3)	
C4	0.38044 (9)	0.90927 (9)	0.44011 (9)	0.0202 (3)	
H4A	0.3360	0.9195	0.4021	0.024*	
C5	0.46187 (9)	0.90041 (9)	0.41113 (8)	0.0200 (3)	
H5A	0.4747	0.9048	0.3541	0.024*	
C6	0.52433 (8)	0.88464 (9)	0.46988 (8)	0.0182 (3)	
C7	0.64171 (9)	0.86485 (9)	0.54132 (9)	0.0184 (3)	
C8	0.73210 (8)	0.85749 (9)	0.56059 (8)	0.0182 (3)	
C9	0.78806 (9)	0.81167 (10)	0.51027 (9)	0.0212 (3)	
H9A	0.7671	0.7838	0.4622	0.025*	
C10	0.87401 (9)	0.80575 (10)	0.52865 (9)	0.0221 (3)	
H10A	0.9118	0.7742	0.4944	0.027*	
C11	0.90130 (8)	0.84735 (9)	0.59813 (9)	0.0196 (3)	
C12	0.84659 (9)	0.89351 (9)	0.64855 (8)	0.0185 (3)	
C13	0.76188 (9)	0.89913 (9)	0.63259 (8)	0.0195 (3)	
H13A	0.7248	0.9297	0.6683	0.023*	
C14	0.27411 (9)	0.91028 (9)	0.55607 (9)	0.0203 (3)	
C15	0.13033 (9)	0.92811 (14)	0.51877 (11)	0.0346 (4)	
H15A	0.1172	0.9892	0.5326	0.042*	
H15B	0.1185	0.8917	0.5677	0.042*	
C16	0.07810 (10)	0.89887 (12)	0.44744 (11)	0.0332 (4)	
H16A	0.0182	0.9039	0.4615	0.050*	
H16B	0.0914	0.8382	0.4346	0.050*	
H16C	0.0904	0.9353	0.3994	0.050*	
C17	0.65550 (9)	0.89081 (10)	0.38489 (8)	0.0205 (3)	
H17A	0.7154	0.9030	0.3976	0.025*	
H17B	0.6316	0.9431	0.3582	0.025*	
C18	0.65105 (9)	0.81489 (10)	0.32436 (9)	0.0239 (3)	
H18A	0.6717	0.7613	0.3515	0.029*	
H18B	0.5918	0.8053	0.3075	0.029*	
C19	0.70487 (10)	0.83430 (11)	0.24854 (9)	0.0270 (3)	
H19A	0.6968	0.7876	0.2074	0.032*	
H19B	0.6859	0.8896	0.2235	0.032*	
C20	0.84682 (9)	0.77241 (10)	0.28605 (9)	0.0241 (3)	
H20A	0.8332	0.7124	0.2839	0.029*	
C21	0.92254 (10)	0.80816 (10)	0.30661 (9)	0.0252 (3)	
H21A	0.9715	0.7759	0.3210	0.030*	
C22	0.84051 (10)	0.91387 (10)	0.28135 (9)	0.0273 (3)	
H22A	0.8188	0.9709	0.2745	0.033*	
C23	0.97877 (9)	0.91055 (11)	0.69681 (9)	0.0248 (3)	
H23A	1.0084	0.9647	0.6811	0.030*	
H23B	1.0066	0.8861	0.7463	0.030*	

### Atomic displacement parameters (Å^2^)

**Table:**

	*U*^11^	*U*^22^	*U*^33^	*U*^12^	*U*^13^	*U*^23^
O1	0.0160 (5)	0.0441 (7)	0.0245 (5)	0.0033 (4)	0.0008 (4)	0.0050 (5)
O2	0.0243 (5)	0.0349 (6)	0.0220 (5)	0.0040 (5)	0.0030 (4)	0.0006 (5)
O3	0.0217 (5)	0.0320 (6)	0.0181 (5)	−0.0026 (4)	−0.0009 (4)	−0.0034 (4)
O4	0.0179 (5)	0.0356 (6)	0.0253 (5)	−0.0013 (4)	−0.0006 (4)	−0.0055 (5)
N1	0.0199 (5)	0.0202 (6)	0.0182 (6)	0.0012 (4)	−0.0008 (4)	−0.0003 (4)
N2	0.0174 (5)	0.0210 (6)	0.0178 (5)	0.0014 (4)	0.0011 (4)	−0.0003 (4)
N3	0.0263 (6)	0.0261 (7)	0.0151 (5)	0.0031 (5)	0.0024 (4)	−0.0009 (5)
N4	0.0328 (7)	0.0283 (7)	0.0276 (7)	−0.0050 (6)	0.0045 (5)	0.0027 (6)
C1	0.0212 (6)	0.0171 (7)	0.0163 (6)	0.0011 (5)	−0.0008 (5)	0.0002 (5)
C2	0.0213 (6)	0.0182 (7)	0.0174 (6)	0.0007 (5)	0.0011 (5)	−0.0007 (5)
C3	0.0193 (6)	0.0179 (7)	0.0199 (7)	0.0012 (5)	0.0011 (5)	−0.0007 (5)
C4	0.0199 (6)	0.0210 (7)	0.0196 (7)	0.0010 (5)	−0.0018 (5)	0.0015 (5)
C5	0.0223 (7)	0.0218 (7)	0.0160 (6)	0.0009 (5)	0.0003 (5)	0.0005 (5)
C6	0.0183 (6)	0.0173 (7)	0.0188 (6)	0.0002 (5)	0.0014 (5)	−0.0009 (5)
C7	0.0209 (6)	0.0160 (7)	0.0184 (6)	0.0010 (5)	−0.0009 (5)	−0.0008 (5)
C8	0.0201 (6)	0.0170 (7)	0.0176 (6)	0.0002 (5)	−0.0010 (5)	0.0020 (5)
C9	0.0220 (6)	0.0197 (7)	0.0220 (7)	0.0011 (5)	−0.0014 (5)	−0.0037 (5)
C10	0.0211 (6)	0.0217 (7)	0.0236 (7)	0.0020 (5)	0.0025 (5)	−0.0027 (6)
C11	0.0172 (6)	0.0205 (7)	0.0212 (7)	−0.0015 (5)	0.0010 (5)	0.0034 (5)
C12	0.0229 (6)	0.0180 (7)	0.0146 (6)	−0.0022 (5)	0.0000 (5)	0.0015 (5)
C13	0.0217 (6)	0.0198 (7)	0.0171 (6)	0.0019 (5)	0.0010 (5)	−0.0002 (5)
C14	0.0206 (6)	0.0172 (7)	0.0230 (7)	0.0010 (5)	0.0000 (5)	0.0002 (5)
C15	0.0174 (7)	0.0565 (12)	0.0300 (9)	0.0047 (7)	0.0029 (6)	0.0029 (8)
C16	0.0223 (7)	0.0453 (10)	0.0320 (9)	−0.0005 (7)	−0.0004 (6)	0.0054 (7)
C17	0.0214 (6)	0.0228 (7)	0.0173 (6)	0.0009 (5)	0.0023 (5)	0.0007 (5)
C18	0.0228 (7)	0.0275 (8)	0.0215 (7)	0.0005 (6)	−0.0025 (5)	−0.0044 (6)
C19	0.0279 (7)	0.0372 (9)	0.0159 (6)	0.0055 (6)	−0.0021 (5)	−0.0030 (6)
C20	0.0296 (7)	0.0215 (7)	0.0213 (7)	0.0032 (6)	0.0015 (6)	−0.0030 (6)
C21	0.0272 (7)	0.0266 (8)	0.0219 (7)	0.0011 (6)	0.0032 (6)	−0.0003 (6)
C22	0.0381 (8)	0.0224 (8)	0.0215 (7)	0.0003 (6)	0.0041 (6)	0.0029 (6)
C23	0.0207 (6)	0.0318 (8)	0.0219 (7)	−0.0028 (6)	−0.0013 (5)	−0.0010 (6)

### Geometric parameters (Å, °)

**Table:**

O1—C14	1.3396 (17)	C8—C13	1.4140 (19)
O1—C15	1.4526 (18)	C9—C10	1.3981 (19)
O2—C14	1.2170 (18)	C9—H9A	0.9500
O3—C12	1.3792 (16)	C10—C11	1.367 (2)
O3—C23	1.4516 (18)	C10—H10A	0.9500
O4—C11	1.3745 (17)	C11—C12	1.3883 (19)
O4—C23	1.4361 (19)	C12—C13	1.3707 (19)
N1—C7	1.3233 (18)	C13—H13A	0.9500
N1—C1	1.3902 (17)	C15—C16	1.493 (2)
N2—C6	1.3793 (17)	C15—H15A	0.9900
N2—C7	1.3889 (18)	C15—H15B	0.9900
N2—C17	1.4613 (17)	C16—H16A	0.9800
N3—C22	1.357 (2)	C16—H16B	0.9800
N3—C20	1.3713 (19)	C16—H16C	0.9800
N3—C19	1.460 (2)	C17—C18	1.529 (2)
N4—C22	1.314 (2)	C17—H17A	0.9900
N4—C21	1.374 (2)	C17—H17B	0.9900
C1—C2	1.3950 (19)	C18—C19	1.527 (2)
C1—C6	1.4055 (19)	C18—H18A	0.9900
C2—C3	1.3930 (19)	C18—H18B	0.9900
C2—H2A	0.9500	C19—H19A	0.9900
C3—C4	1.414 (2)	C19—H19B	0.9900
C3—C14	1.4827 (19)	C20—C21	1.362 (2)
C4—C5	1.3808 (19)	C20—H20A	0.9500
C4—H4A	0.9500	C21—H21A	0.9500
C5—C6	1.3960 (19)	C22—H22A	0.9500
C5—H5A	0.9500	C23—H23A	0.9900
C7—C8	1.4714 (18)	C23—H23B	0.9900
C8—C9	1.3971 (19)		
			
C14—O1—C15	116.47 (12)	C8—C13—H13A	121.5
C12—O3—C23	105.42 (11)	O2—C14—O1	123.34 (13)
C11—O4—C23	105.71 (11)	O2—C14—C3	124.47 (13)
C7—N1—C1	104.66 (11)	O1—C14—C3	112.18 (12)
C6—N2—C7	106.21 (11)	O1—C15—C16	107.36 (14)
C6—N2—C17	123.07 (12)	O1—C15—H15A	110.2
C7—N2—C17	129.61 (11)	C16—C15—H15A	110.2
C22—N3—C20	106.20 (13)	O1—C15—H15B	110.2
C22—N3—C19	127.97 (13)	C16—C15—H15B	110.2
C20—N3—C19	125.67 (13)	H15A—C15—H15B	108.5
C22—N4—C21	104.39 (13)	C15—C16—H16A	109.5
N1—C1—C2	130.07 (13)	C15—C16—H16B	109.5
N1—C1—C6	110.32 (12)	H16A—C16—H16B	109.5
C2—C1—C6	119.58 (12)	C15—C16—H16C	109.5
C3—C2—C1	118.01 (13)	H16A—C16—H16C	109.5
C3—C2—H2A	121.0	H16B—C16—H16C	109.5
C1—C2—H2A	121.0	N2—C17—C18	113.51 (12)
C2—C3—C4	121.29 (13)	N2—C17—H17A	108.9
C2—C3—C14	117.30 (12)	C18—C17—H17A	108.9
C4—C3—C14	121.40 (12)	N2—C17—H17B	108.9
C5—C4—C3	121.39 (13)	C18—C17—H17B	108.9
C5—C4—H4A	119.3	H17A—C17—H17B	107.7
C3—C4—H4A	119.3	C19—C18—C17	110.03 (13)
C4—C5—C6	116.60 (13)	C19—C18—H18A	109.7
C4—C5—H5A	121.7	C17—C18—H18A	109.7
C6—C5—H5A	121.7	C19—C18—H18B	109.7
N2—C6—C5	131.09 (13)	C17—C18—H18B	109.7
N2—C6—C1	105.74 (12)	H18A—C18—H18B	108.2
C5—C6—C1	123.12 (13)	N3—C19—C18	111.80 (12)
N1—C7—N2	113.06 (12)	N3—C19—H19A	109.3
N1—C7—C8	123.27 (12)	C18—C19—H19A	109.3
N2—C7—C8	123.62 (12)	N3—C19—H19B	109.3
C9—C8—C13	120.01 (13)	C18—C19—H19B	109.3
C9—C8—C7	122.22 (12)	H19A—C19—H19B	107.9
C13—C8—C7	117.77 (12)	C21—C20—N3	105.91 (14)
C8—C9—C10	121.81 (13)	C21—C20—H20A	127.0
C8—C9—H9A	119.1	N3—C20—H20A	127.0
C10—C9—H9A	119.1	C20—C21—N4	110.72 (14)
C11—C10—C9	117.00 (13)	C20—C21—H21A	124.6
C11—C10—H10A	121.5	N4—C21—H21A	124.6
C9—C10—H10A	121.5	N4—C22—N3	112.77 (14)
C10—C11—O4	127.96 (13)	N4—C22—H22A	123.6
C10—C11—C12	121.89 (13)	N3—C22—H22A	123.6
O4—C11—C12	110.12 (12)	O4—C23—O3	107.33 (11)
C13—C12—O3	128.07 (13)	O4—C23—H23A	110.2
C13—C12—C11	122.22 (13)	O3—C23—H23A	110.2
O3—C12—C11	109.69 (12)	O4—C23—H23B	110.2
C12—C13—C8	117.05 (13)	O3—C23—H23B	110.2
C12—C13—H13A	121.5	H23A—C23—H23B	108.5
			
C7—N1—C1—C2	−177.49 (15)	C23—O4—C11—C10	172.62 (15)
C7—N1—C1—C6	0.61 (16)	C23—O4—C11—C12	−9.19 (16)
N1—C1—C2—C3	178.57 (14)	C23—O3—C12—C13	−174.54 (15)
C6—C1—C2—C3	0.6 (2)	C23—O3—C12—C11	6.74 (15)
C1—C2—C3—C4	−1.1 (2)	C10—C11—C12—C13	1.0 (2)
C1—C2—C3—C14	177.48 (13)	O4—C11—C12—C13	−177.28 (13)
C2—C3—C4—C5	0.5 (2)	C10—C11—C12—O3	179.84 (13)
C14—C3—C4—C5	−178.07 (13)	O4—C11—C12—O3	1.53 (16)
C3—C4—C5—C6	0.7 (2)	O3—C12—C13—C8	179.77 (13)
C7—N2—C6—C5	176.99 (15)	C11—C12—C13—C8	−1.7 (2)
C17—N2—C6—C5	8.0 (2)	C9—C8—C13—C12	1.3 (2)
C7—N2—C6—C1	−0.47 (15)	C7—C8—C13—C12	−178.39 (12)
C17—N2—C6—C1	−169.43 (12)	C15—O1—C14—O2	1.4 (2)
C4—C5—C6—N2	−178.25 (14)	C15—O1—C14—C3	−179.31 (13)
C4—C5—C6—C1	−1.2 (2)	C2—C3—C14—O2	2.2 (2)
N1—C1—C6—N2	−0.08 (16)	C4—C3—C14—O2	−179.15 (14)
C2—C1—C6—N2	178.25 (12)	C2—C3—C14—O1	−177.08 (13)
N1—C1—C6—C5	−177.79 (13)	C4—C3—C14—O1	1.5 (2)
C2—C1—C6—C5	0.5 (2)	C14—O1—C15—C16	−155.28 (14)
C1—N1—C7—N2	−0.93 (16)	C6—N2—C17—C18	−81.03 (16)
C1—N1—C7—C8	176.36 (13)	C7—N2—C17—C18	112.77 (16)
C6—N2—C7—N1	0.91 (16)	N2—C17—C18—C19	−175.90 (12)
C17—N2—C7—N1	168.90 (13)	C22—N3—C19—C18	−99.29 (17)
C6—N2—C7—C8	−176.38 (13)	C20—N3—C19—C18	75.53 (18)
C17—N2—C7—C8	−8.4 (2)	C17—C18—C19—N3	64.76 (16)
N1—C7—C8—C9	144.90 (15)	C22—N3—C20—C21	−0.74 (16)
N2—C7—C8—C9	−38.1 (2)	C19—N3—C20—C21	−176.49 (13)
N1—C7—C8—C13	−35.4 (2)	N3—C20—C21—N4	0.52 (17)
N2—C7—C8—C13	141.59 (14)	C22—N4—C21—C20	−0.09 (18)
C13—C8—C9—C10	−0.3 (2)	C21—N4—C22—N3	−0.41 (17)
C7—C8—C9—C10	179.35 (13)	C20—N3—C22—N4	0.74 (17)
C8—C9—C10—C11	−0.4 (2)	C19—N3—C22—N4	176.37 (13)
C9—C10—C11—O4	178.02 (14)	C11—O4—C23—O3	13.14 (15)
C9—C10—C11—C12	0.0 (2)	C12—O3—C23—O4	−12.24 (15)

### Hydrogen-bond geometry (Å, °)

**Table:**

*Cg*1 and *Cg*4 are the centroids of C11/C12/O3/C23/O4 and C1–C6 rings, respectively.

**Table:**

*D*—H···*A*	*D*—H	H···*A*	*D*···*A*	*D*—H···*A*
C2—H2A···O3^i^	0.95	2.48	3.3922 (17)	162
C15—H15B···O4^ii^	0.99	2.49	3.213 (2)	130
C23—H23A···N4^iii^	0.99	2.43	3.379 (2)	159
C10—H10A···Cg4^iv^	0.95	2.65	3.3005 (16)	126
C16—H16C···Cg1^v^	0.98	2.91	3.7282 (19)	142

Symmetry codes: (i) *x*−1/2, *y*, −*z*+3/2; (ii) *x*−1, *y*, *z*; (iii) −*x*+2, −*y*+2, −*z*+1; (iv) −*x*, *y*+3/2, −*z*+3/2; (v) −*x*+1, −*y*+2, −*z*+1.
